# Vitamin D status in active and inactive noninfectious uveitis - data from a reference university hospital in Rio de Janeiro, Brazil

**DOI:** 10.5935/0004-2749.2023-0141

**Published:** 2024-03-27

**Authors:** Henrique Maciel Vieira de Moraes, Juliana Rocha de Mendonça da Silva, Milena Ribeiro Rangel, Marcelle Raschik Riche, Haroldo Vieira de Moraes Junior

**Affiliations:** 1 Serviço de Oftalmologia, Hospital Universitário Clementino Fraga Filho, Universidade Federal do Rio de Janeiro, Rio de Janeiro, RJ, Brazil

**Keywords:** Vitamin D, 25-hydroxyvitamin D, Uveitis, Vitamin D deficiency, Immunity, Eye/immunology

## Abstract

**Purpose:**

This study aimed to investigate the correlation between serum vitamin D levels and disease activity in patients with noninfectious uveitis.

**Methods:**

We conducted a prospective case-control study, assessing 51 patients with noninfectious uveitis, categorized into active (n=22) and inactive (n=29) groups, along with 51 healthy controls. Serum 25-hydroxy vitamin D [25(OH)D] levels were measured. The uveitis group also completed a questionnaire regarding sunlight exposure habits and vitamin D supplementation.

**Results:**

Patients with inflammation-related uveitis exhibited low serum 25(OH)D levels in 68% of cases. The median 25(OH)D level in patients with active uveitis was 17.8 ng/mL (interquartile range [IQR], 15-21 ng/mL), significantly lower compared to the 31.7 ng/mL (IQR, 25-39 ng/mL) in patients with inactive uveitis (p<0.001) and the 27 ng/mL (IQR, 23-31 ng/mL) in the Control Group (p<0.001). Significantly, nearly all patients with uveitis taking vitamin D supplementation were in the Inactive Group (p<0.005). Moreover, reduced sunlight exposure was associated with active uveitis (p<0.003). Furthermore, patients with 25(OH)D levels below 20 ng/mL had ten times higher odds of developing active uveitis (p=0.001).

**Conclusions:**

This study revealed a prevalent 25(OH)D deficiency among patients with noninfectious uveitis and suggested a link between low 25(OH)D levels and disease activity. To prevent future episodes of intraocular inflammation, vitamin D supplementation and controlled sunlight exposure could be viable options.

## INTRODUCTION

Uveitis stands as a leading global cause of irreversible blindness, often posing a formidable diagnostic and therapeutic challenge for ophthalmologists^(1)^. Specifically, noninfectious immune-mediated uveitis, the most prevalent type in developed countries, predominantly afflicts young adults aged 20 to 50 years. Moreover, treatments for this condition are seldom curative and frequently carry systemic and vision-threatening side effects^(2)^. The chronic and recurrent nature of these intraocular inflammations inflicts a substantial socioeconomic burden and significantly diminishes the quality of life^(3)^.

Vitamin D, primarily recognized for its role in calcium homeostasis, is an essential steroid hormone. In recent years, it has unveiled additional functions, including immunomodulatory and anti-inflammatory properties^(4)^. Furthermore, vitamin D deficiency has been associated with numerous autoimmune diseases, such as spondylarthritis, inflammatory bowel disease, juvenile idiopathic arthritis (JIA), and systemic lupus erythematosus, all closely associated with uveitis^(5^-^8)^.

Understanding the pivotal role of vitamin D in both the innate and adaptive arms of the immune system, especially its potential to promote immune tolerance, is fundamental in elucidating its protective potential against the development of autoimmune diseases^(9^,^10)^. In noninfectious immune-mediated uveitis, tissue damage results from T lymphocyte responses to uveal tract antigens. Notably, the expression of the vitamin D receptor (VDR) has been identified in many immune cell types and tissues, including those in the ocular domain. Beyond regulating immune cell differentiation and proliferation, VDR can activate inactive vitamin D metabolites into calcitriol. By elevating T helper-2 cytokines and reducing T helper-1 cytokines levels, calcitriol enhances humoral-mediated immunity over cell-mediated immunity^(11^,^12)^.

In experimental autoimmune uveitis, the oral administration of calcitriol has shown promise in preventing intraocular inflammation, reversing the disease process and mitigating the associated immunological response. By suppressing interleukin (IL)-17 production, a factor implicated in the pathogenesis of many autoimmune conditions, including uveitis, and inhibiting T helper-17 responses, VDR agonists may hold therapeutic potential for uveitis treatment^(13)^.

Furthermore, associations have been reported between low vitamin D levels and conditions such as acute anterior uveitis, VKH-associated uveitis, and sarcoidosis--associated uveitis^(14^-^16)^. This study’s objective is to inves-tigate and compare vitamin D levels and sunlight exposure habits among patients with noninfectious uveitis with varying disease activity, alongside healthy controls, at a referral center in Rio de Janeiro, Brazil.

## METHODS

This case-control study was approved by the institutional ethics committee of the Federal University of Rio de Janeiro (UFRJ) and conformed to the principles outlined in the Declaration of Helsinki^(17)^. Prior to their enrollment in the study, written informed consent was obtained from all participants.

The case group consisted of 51 adults aged 18 years and older, consecutively recruited between June 2022 and March 2023, from the ophthalmology department of a public eye care reference service (Clementino Fraga Filho University Hospital- UFRJ, Brazil). These individuals were categorized as having active or inactive noninfectious uveitis. Exclusion criteria encompassed individuals with suspected or confirmed infectious uveitis, traumatic uveitis, drug-induced uveitis, or masquerade syndrome. Furthermore, patients with systemic illnesses, those taking medications that could alter vitamin D levels, pregnant women, and individuals with conditions impacting vitamin D levels were excluded. A control group, matched in terms of age and sex, was constituted of 51 patients without any history of eye inflammation and had recorded serum 25(OH)D levels, also known as calcidiol, along with a normal ophthalmological examination apart from refractive errors.

Participants underwent a comprehensive ophthalmological examination, which encompassed detailed anamnesis, ocular ectoscopy, best-corrected visual acuity measurement, tonometry, anterior and posterior biomicroscopy, indirect ophthalmoscopy, and supplementary evaluations when necessary, including retinal imaging. The diagnosis of uveitis was established based on the criteria set forth by the Standardized Uveitis Nomenclature (SUN) as defined by the International Uveitis Study Group^(18)^. Uveitis was defined as active if slit-lamp examination revealed uveitis activity within the 30 days preceding the 25(OH)D blood test. Anatomically, anterior uveitis was considered active if it exhibited more than 0.5+ cells in the anterior chamber or 1+ flare, and intermediate uveitis if it presented more than 0.5+ vitreous cells, and posterior uveitis was regarded as active when chorioretinal inflammation was evident^(19)^.

A comprehensive protocol form was used to collect data for each case, including information on age, gender, clinical history, associated systemic disease, uveitis acti-vity, anatomical uveitis diagnoses, previous intraocular inflammation (first episode or relapse), and identifiable uveitis syndrome.

Following the ophthalmologic examination, patients underwent blood tests to measure serum 25(OH)D levels using electrochemiluminescence analysis. Calcidiol deficiency was defined as a serum 25(OH)D concentration of less than 20 ng/mL in accordance with the guidelines of the Brazilian Society of Endocrinology and Metabolism^(20)^.

Considering the significant relationship between vitamin D synthesis and daily sun exposure habits, participants were requested to respond to a simple questionnaire featuring multiple-choice questions, derived from the Quantitative Assessment of Solar UV Exposure for Vitamin D Synthesis in Australian Adults (AusD) Study^(21)^. This questionnaire covered the length of time spent in sunlight (less than 30 min, 30-60 min, 60-120 min, or more), the frequency of using sun exposure protection methods (such as hat, sunscreen, and long-sleeved shirt) (never, less than half the time, more than half the time, always), and the history of vitamin D supplementation (yes, no). Considering that skin pigmentation can influence the synthesis of vitamin D mediated by ultraviolet radiation, participants were asked about how their skin responds to sun exposure using the Fitzpatrick scale (Fitzpatrick skin types I-III represent lighter-skinned individuals and types IV-VI represent darker-skinned individuals)^(21)^.

All study participants were residents of Rio de Janeiro, located in the southeast region of Brazil. As the second largest metropolis in the country, with an estimated population of over six million in 2021, the city boasts a nearly 200-kilometer coastline and experiences a predominately semi-humid tropical climate. The average temperature is around 24°C, and the city enjoys approximately 4,380 hours of sunlight annually, according to the National Weather Prevision Center and Climate Studies.

### Statistical analysis

Categorical variables were presented as numbers and frequency (%), while numerical variables were expressed as medians with interquartile ranges spanning the 25^th^ and 75^th^ percentiles. Comparisons of age and serum 25(OH)D levels among groups were performed using the Mann-Whitney test for two-group comparisons or the Kruskal-Wallis test for three-group comparisons. Furthermore, the Wilcoxon test was used to compare the serum 25(OH)D levels paired by sex and age between control and case groups. Frequency distribution comparisons among different groups were assessed using the chi-square test. To calculate the odds ratio (OR), logistic was conducted, with a 95% confidence interval (95% CI) considered. Statistical analysis was executed using SPSS version 21.

## RESULTS

This study encompassed a total of 51 patients with noninfectious uveitis, with an age and sex-matched Control Group comprising 51 healthy individuals. Within the uveitis cohort, 22 patients exhibited active uveitis and 29 had inactive uveitis. The median age was 51 years (37-55 years) for patients with active uveitis and 49 years (38-59 years) for those with inactive uveitis. Notably, although not statistically significant (p=0.380), a higher proportion of females (48.5%) presented with intraocular inflammation activity compared to males (33%). A detailed overview of the demographic and disease characteristics of patients grouped according to their uveitis inflammatory status compared with healthy controls is provided in table 1.

**Table 1:** Demographic and disease characteristics of the study participants

	Active Uveitis	Inactive Uveitis	Healthy controls	p-value (Active uveitis vs. Inactive)
Number of patients	22	29	51	
Age (years), median (IQR)	51 (37-55)	49 (38-59)	50 (34-58)	0.802
Male, n (%)	6 (27.3)	12 (41.4)	18 (35.3)	0.380
Anatomic classification, n (%)				0.823
Anterior	11 (50)	11 (37.9)		
Intermediate	4 (18.2)	06 (20.7)		
Posterior	2 (9.1)	03 (10.3)		
Panuveitis	5 (22.7)	9 (31)		
Etiological diagnosis, n (%)				0.779
Behçet disease	3 (13.6)	5 (17.2)		
Bowel disease	1 (4.5)	1 (03.4)		
Ankylosing spondylitis	3 (13.6)	3 (10.3)		
HLA B27 +	0	2 (06.9)		
Idiopathic	9 (40.9)	7 (24.1)		
SLE	1 (4.5)	1 (03.4)		
Sarcoidosis	0	1 (03.4)		
VKH syndrome	5 (22.7)	9 (031)		
First presentation, n (%)	8 (36.4)	0		<0.001
Vitamin D intake, n (%)	1 (4.5)	11 (37.9)		0.005

IQR= interquartile range; SEL= systemic lupus erythematous; VKH= Vogt-Koyanagi--Harada.

The predominant uveitis syndromes reported in both active and inactive groups were Vogt-Koyanagi-Harada syndrome (n=14) and Behçet’s disease (n=9). Systemic associations were documented and included inflammatory bowel disease, HLA-B27-related disease, systemic lupus erythematosus, and sarcoidosis. Among the anatomical diagnoses, anterior uveitis was the most common, with 11 cases in each of the active and inactive groups.

Vitamin D supplementation was reported by almost 40% of patients with inactive uveitis (n=11), while only one patient in the active group reported supplementation. Notably, a majority of individuals in the active Uveitis Group had experienced previous episodes of intraocular inflammation (n=14, 63.6%).

**Figure 1:**
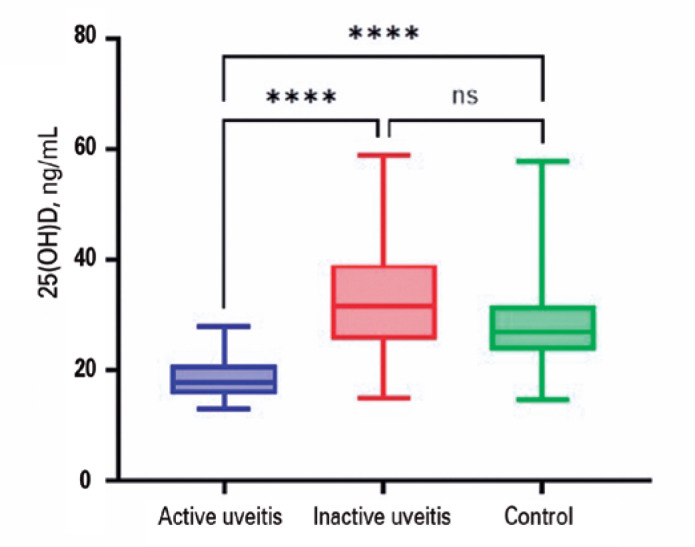
Distribution of serum 25 (OH) D levels (ng/mL), in patients with active uveitis, inactive uveitis and healthy controls.

According to Table 2 and Figure 1, the median serum 25(OH)D level was 17.8 ng/mL (IQR, 15-21ng/mL) in the active group, contrasting with 31.7 ng/mL (IQR, 25-39 ng/mL) in the Inactive Group, and 27 ng/mL (IQR, 23-31 ng/mL) in healthy controls (p<0.001). While both the inactive and control groups exhibited median values above the deficient range of 20 ng/mL and reported similar percentages of inadequate 25(OH)D levels (17.2% and 11.8%, respectively), a significant 68.2% of patients with active uveitis had serum 25(OH)D levels below 20 ng/mL (p<0.001). Notably, there was a significant reduction in the odds of manifesting uveitis activity when serum 25(OH)D levels exceeded 20 ng/mL (OR=0.09; CI 95%=0.02; 0.36).

**Table 2:** Participants median serum vitamin D levels

25(OH)D serum level	Active uveitis	Inactive uveitis	Healthy controls	p-value (active vs. inactive)
Median (ng/mL), (IQR)	17.8 (15-21)	31.7 (25-39)	27 (23-31)	<0.001
Deficient (<20 ng/mL), n (%)	15 (68.2)	5 (17.2)	6 (11.8)	<0.001

IQR= interquartile range.

Responses related to sunlight exposure habits were compared between the active and inactive uveitis groups. Patients with active uveitis exhibited lower levels of sunlight exposure compared to their inactive uveitis counterparts (p<0.001). However, both groups displayed similar behaviors regarding solar protection (p=0.291). Furthermore, there was no statistically significant association between skin type and tanning ability with uveitis inflammatory activity (p=0.719) (Table 3).

**Table 3:** Associations among skin phototypes, sunlight exposure, and uveitis activity

	Active uveitis	Inactive uveitis	p-value
Skin phototypes, n (%)			0.719
Type III	9 (40.9)	11 (37.9)	
Type IV	4 (18.2)	08 (27.6)	
Type V	7 (31.8)	06 (20.7)	
Type VI	2 (09.1)	04 (13.8)	
Time spent in sunlight, n (%)			<0.001
<30 minutes	06 (27.3)	0	
30-60 minutes	14 (63.6)	9 (31)	
60-120 minutes	2 (9.1)	19 (65.5)	
>120 minutes	0	1 (3.4)	
Sun protection, n (%)			0.291
Never	4 (18.2)	11 (37.9)	
Less than half the time	11 (50)	14 (48.3)	
More than half the time	6 (27.3)	03 (10.3)	
Always	1 (4.5)	01 (03.4)	

Similar correlations were investigated within a subgroup of patients with uveitis with 25(OH)D levels lower than 20 ng/mL, encompassing 15 with active uveitis and 5 with inactive uveitis (Table 4). While participants with active disease activity reported less sunlight exposure compared to their inactive counterparts, this association did not exhibit statistical significance.

**Table 4:** Associations among skin phototypes, sunlight exposure, and uveitis activity in participants with 25(OH)D deficiency (<20 ng/mL)

	Active uveitis (n=15)	Inactive uveitis (n=5)	p-value
Skin phototypes, n (%)			0.962
Type III	5 (33.3)	2 (40)	
Type IV	4 (26.7)	1 (20)	
Type V	4 (26.7)	1 (20)	
Type VI	2 (13.3)	1 (20)	
Time spent in sunlight, n (%)			0.031
<30 minutes	3 (20)	0	
30-60 minutes	11 (73.3)	2 (40)	
60-120 minutes	1 (6.7)	3 (60)	
>120 minutes	0	0	
Sun protection, n (%)			0.588
Never	2 (13.3)	2 (40)	
Less than half the time	7 (46.7)	2 (40)	
More than half the time	5 (33.3)	1 (20)	
Always	1 (6.7)	0	

## DISCUSSION

This study was conducted at a specialized reference center in the state of Rio de Janeiro, reflecting a com-prehensive overview of the most complicated and challenging uveitis cases in the region. Notably, we emphasize the inclusion of patients with uveitis of different etiologies and anatomical classifications, rendering the findings more broadly applicable to the population.

The consecutive recruitment of patients was adopted to mitigate the potential selection bias inherent in retrospective studies. Moreover, the inclusion of healthy controls residing in the same geographic region as the patients with uveitis, exposed to similar climate conditions, adds to the robustness of our findings.

Our results demonstrated that patients with active uveitis displayed significantly lower levels of serum 25(OH)D compared to patients with inactive uveitis and healthy controls. These findings corroborate with previous literature, wherein a relationship between hypovitaminosis D and noninfectious uveitis has been consistently documented^(22^-^25)^. Table 5 presents a summary of clinical evidence from recent global studies, including the results of this study, along with their respective study populations.

**Table 5:** Clinical characteristics from studies on the vitamin D status and uveites

	Moraes et al. RJ, Brazil 2023	Grotting et al.^(23)^USA 2016	Sengler et al.^(6)^ Germany 2018	Llop et al.^(24)^USA 2019	Chiu et al.^(25)^Australia 2019
**Patients**	Active vs. inactive noninfectious uveitis and controls	Noninfectious autoimune anterior uveitis vs. controls	JIA patients with and without uveitis	Noninfectious uveitis or scleritis vs. controls	Active vs. inactive noninfectious uveitis
**Study**	Observational case-control	Retrospective case-control	Retrospective case-control	Retrospective case-control	Observational case-control
**Results**	Low vitamin D levels associated with uveitis activity	Low vitamin D levels associated with risk of noninfectious anterior uveitis	Low vitamin D levels associated with risk of uveitis in JIA patients	Low vitamin D levels associated with risk of noninfectious uveitis and scleritis	Low vitamin D levels and sun deprivation associated with uveitis activity.

RJ= Rio de Janeiro; USA= United States of America; JIA= juvenile idiophatic arthritis.

In our sample, patients with active uveitis exhibited 25(OH)D values that were 44% lower than those without disease activity and 34% lower than the control population. Notably, nearly 70% of patients with active uveitis had 25(OH)D levels below 20 ng/mL, in stark contrast to 17% of patients with inactive uveitis and 12% among those without a history of uveitis. Moreover, none of the patients with active uveitis had 25(OH)D levels exceeding 30 ng/mL, while 59% of patients without disease activity surpassed this threshold. These findings suggest that higher 25(OH)D levels may confer benefits in controlling intraocular inflammation. Importantly, the Endocrine Society Practice Guideline advocated for 25(OH)D levels of ≥30 ng/mL, categorizing levels of 21-29 ng/mL and ≤20 ng/mL as insufficiency and deficiency, respectively^(22)^.

Vitamin D can be obtained from dietary sources, but its main source is the body’s capacity for endogenous synthesis. The synthesis of active vitamin D begins in the skin upon exposure to ultraviolet B (UVB) sunlight^(26)^. Consequently, daily habits related to sun exposure, vitamin D supplementation, and skin pigmentation hold a significant influence over serum 25(OH)D levels^(27)^. In our study, participants with active uveitis reported reduced sun exposure and a greater adherence to UVB radiation protection measures. These trends persisted when focusing on patients with serum 25(OH)D deficiency. Moreover, only one individual with active uveitis reported vitamin D supplementation, in contrast to almost 40% of participants in the inactive group. This indicates that supplementation may represent a crucial means of attaining adequate calcidiol levels, even in regions characterized by abundant sunlight throughout the year. Our results are consistent with previous assessments on uveitis and sun exposure, both internationally and recently in Brazil^(25^,^28)^.

However, owing to the observational design of our study, we can only infer an association between low serum 25(OH)D levels and disease activity, while the possibility of reverse causation must be considered. Patients with active uveitis may experience photophobia, leading them to reduce sun exposure. Nonetheless, it is plausible that both groups, active and inactive, may have reduced their sun exposure to some extent due to the presence or history of uveitis.

Although we believe vitamin D deficiency is associated with ocular disease, our understanding of the pathogenic mechanisms remains limited. Additionally, it is worth noting that hypovitaminosis D is a common finding in certain granulomatous diseases, such as sarcoidosis, which is known to be associated with hypercalcemia and increased calcitriol production. Thus, routine assessment of vitamin D levels is recommended^(16)^.

Laboratory testing for the diagnosis of hypovitaminosis D involves a simple blood sample, rendering its management economically viable and efficient^(29)^. As a future perspective, interventional studies are warranted to ascertain whether vitamin D supplementation, aimed at achieving adequate 25(OH)D levels, possesses the potential to prevent the development of noninfectious uveitis and modify its course, thereby serving as an adjuvant treatment.
